# Fisetin Promotes Hair Growth by Augmenting TERT Expression

**DOI:** 10.3389/fcell.2020.566617

**Published:** 2020-10-15

**Authors:** Chisato Kubo, Mizuki Ogawa, Norihisa Uehara, Yoshinori Katakura

**Affiliations:** ^1^Laboratory of Cellular Regulation Technology, Department of Bioscience and Biotechnology, Graduate School of Bioresources and Bioenvironmental Sciences, Kyushu University, Fukuoka, Japan; ^2^Department of Molecular Cell Biology and Oral Anatomy, Faculty of Dental Science, Kyushu University, Fukuoka, Japan; ^3^Laboratory of Cellular Regulation Technology, Department of Bioscience and Biotechnology, Faculty of Agriculture, Kyushu University, Fukuoka, Japan

**Keywords:** TERT, hair-growth, polyphenol, fisetin, resveratrol, HaCaT, C57BL/6

## Abstract

Although thinning hair and alopecia are not recognized as severe diseases, hair loss has implications for mental health and quality of life; therefore, a large number of studies have been carried out to develop novel hair growth agents. In the present study, we aimed to examine the potential of telomerase reverse transcriptase (TERT), because *TERT* overexpression in skin activates resting hair follicle bulge stem cells, which triggers initiation of a new hair follicle growth phase and promotes hair synthesis. To this end, we screened polyphenols that activate *TERT* expression in keratinocytes, and identified resveratrol and fisetin as strong *hTERT*-augmenting compounds. These polyphenols also regulated the gene expression of cytokines such as *IGF-1* and *KGF*, which activate the β-catenin pathway, and *TGF-*β*1*, which plays an important role in maintaining the niche of hair follicle stem cells, thus are thought to play roles in promoting hair growth. We additionally showed that these polyphenols, especially fisetin, promoted hair growth from the shaved dorsal skin of mice, which suggests that these polyphenols activate the transition from telogen to anagen phase. Histological studies indicated that the dorsal skin of mice treated with these polyphenols contained numerous hair follicles and was thickened compared with that in control mice. Furthermore, on the dorsal skin of mice treated with resveratrol and fisetin, a number of proliferating cells (Ki67^+^ cells) were observed around the hair papilla. These results suggest that resveratrol and fisetin induce a shift from telogen to anagen in the hair follicle by inducing proliferation of hair follicle bulge stem cells, thus promoting hair growth.

## Introduction

Various methods for the treatment of hair thinning and alopecia have been developed. Among them, the use of medicinal compounds has been shown to drastically improve hair growth. Two well-known compounds, finasteride and minoxidil, are used to improve thin hair and alopecia. Finasteride, a synthetic 5-α-reductase inhibitor, is used to treat alopecia by suppressing male hormones, but is reported to cause sexual dysfunction (Shen et al., 2018). Minoxidil was originally used as an antihypertensive medication, but is now used as a topical formulation for promoting hair growth. Despite its widespread use, the precise mechanisms of action of minoxidil are not fully understood (Suchonwanit et al., 2019). As a result, in this study, we focused on telomerase reverse transcriptase (TERT) as a novel target for the induction of hair growth.

TERT, the protein component of telomerase, maintains telomere length, and is activated in stem cells, progenitor cells, and cancer cells. Sarin et al. (2005) reported that conditional transgenic induction of *TERT* in the mouse skin epithelium caused a rapid transition from telogen (the resting phase of the hair follicle cycle) to anagen (the active phase), thereby promoting robust hair growth. *TERT* overexpression induced the proliferation of stem cells in the hair follicle bulge region, resulting in histological changes around the hair follicle and subsequent hair growth. Choi et al. (2008) also showed that TERT in skin keratinocytes facilitated the activation of resting hair follicle stem cells, which triggers the initiation of a new hair follicle growth phase and promoting hair synthesis. These reports indicated that augmentation of *TERT* in skin keratinocytes can be a novel target for hair growth promotion, suggesting that original hair growth promoting compounds with a distinct point of action from finasteride and minoxidil can be found. In the present study, we used a novel screening system for compounds that activate the human *TERT* (*hTERT*) promoter in keratinocytes in order to identify candidates that promote hair growth.

To date, we have developed several screening systems based on the same concept. The CMV promoter region of the *EGFP* expression vector was replaced with the promoter of the gene of interest and introduced into tissue-derived cell lines. These recombinant cells were treated with food components, polyphenols, and compounds, and changes in EGFP fluorescence were monitored using an imaging cytometer (IN Cell Analyzer 1000) (Harada et al., 2016; Zhao et al., 2016; Chong et al., 2019). We successfully identified promoter-activating polyphenols and lactic acid bacteria. In the present study, we used a similar system, in which the EGFP reporter vector under the control of the *hTERT* promoter was introduced into the human keratinocyte cell line, HaCaT, and successfully identified several polyphenols that augmented *hTERT* expression. We sought to investigate whether these *hTERT*-augmenting polyphenols promote hair growth, and to clarify the molecular mechanisms underlying their hair-growth-promoting effects.

## Materials and Methods

### Cell Line

The HaCaT human keratinocyte cell line (Riken Bioresource Center, Tsukuba, Japan) was cultured in Dulbecco’s Modified Eagle’s Medium (DMEM; Nissui, Tokyo, Japan) supplemented with 10% fetal bovine serum (FBS; Life Technologies, Gaithersburg, MD, United States) at 37°C in a 5% CO_2_ atmosphere.

### Screening System for Polyphenols That Activate the hTERT Promoter

The human *hTERT* promoter (−298 to −25) was cloned into pEGFP-C3 (TaKaRa, Shiga, Japan), whose CMV promoter was removed by *Ase*I and *Nhe*I digestion (Fujiki et al., 2007). The resulting plasmid (hTERTp-EGFP) was transduced into HaCaT cells [HaCaT (hTERTp-EGFP)]. Changes in the EGFP fluorescence derived from hTERTp-EGFP were monitored using an IN Cell Analyzer 1000 (GE Healthcare, Amersham Place, United Kingdom) (Zhao et al., 2016; Chong et al., 2019).

### Quantitative Reverse Transcription-Polymerase Chain Reaction

RNA was prepared from cells using the High Pure RNA Isolation kit (Roche Diagnostics GmbH, Mannheim, Germany) and from skin samples using the RNeasy Fibrous Tissue Mini Kit (Qiagen, Hilden, Germany) as described in the manufacturers’ protocols. cDNA was prepared using the SuperScript IV Reverse Transcriptase (Thermo Fisher Scientific KK, Tokyo, Japan), as described previously (Sugihara et al., 2019). Quantitative reverse transcription-polymerase chain reaction (qRT-PCR) was performed using the Thunderbird SYBR qPCR mix (Toyobo, Osaka, Japan) and Thermal Cycler Dice Real Time System TP-800 (Takara). Samples were analyzed in triplicate, and gene expression levels were normalized to the corresponding β-actin level. The PCR primer sequences used were as follows: human β*-actin* forward primer 5′-*TGGCACCCAGCACAATGAA*-3′ and reverse primer 5′-*CTAAGTCATAGTCCGCCTAGAAGCA*-3′: human *AXIN2* forward primer 5′-TGGTGCCCTACCATTGACACA-3′ and reverse primer 5′-TGGTCAACCCTCAAGACCTTTAAGA-3′: *hTERT* forward primer 5′-*CGTACAGGTTTCACGCATGTG*-3′ and reverse primer 5′-*ATGACGCGCAGGAAAAATG*-3′: *IGF-1* forward primer 5′-*TCACCTTCACCAGCTCTGCC*-3′ and reverse primer 5′-*AAGCCCCTGTCTCCACACAC*-3′: *KGF* forward primer 5′-*GGACACACAACGGAGGGGAA*-3′ and reverse primer 5′-*TGCCATAGGAAGAAGTGGGCT*-3′: *TGF-*β*1* forward primer 5′-*AACCGGCCTTTCCTGCTTCT*-3′ and reverse primer 5′-*ACGCAGCAGTTCTTCTCCGT*-3′: mouse β*-actin* forward primer 5′-*GAGGTCTTTACGGATGTCAAC*-3′ and reverse primer 5′-*GGCCAGGTCATCACTATTG*-3′: *mTERT* forward primer 5′-*CAGCCATACATGGGCCAGTTC*-3′ and reverse primer 5′-*ACAGGCTGCTGCTGCTCTCA*-3′: mouse β*-catenin* forward primer 5′-GCTGCTGTCCTATTCCGAATGTC-3′ and reverse primer 5′- GGCACCAATGTCCAGTCCAA-3′.

### Luciferase Assay

A reporter containing TCF/LEF elements (M50 Super 8 × TOPFlash) (Veeman et al., 2003) (Addgene, Cambridge, MA, United States) was used as the reporter vector (Harada et al., 2016). The luciferase assay was performed using the Dual-Luciferase Reporter Assay System (Promega, Madison, WI, United States), as described previously (Yamashita et al., 2014).

### Western Blot

Cell lysates were prepared using NP-40 lysis buffer (0.5% Non-idet P-40, 5 mM EDTA, 2 mM Na_3_VO_4_, 10 mM Tris-HCl (pH 7.6), 150 mM NaCl, 5 mg/mL aprotinin, 1 mM PMSF). Protein concentration was determined using the Protein Assay Dye (Bio-Rad Laboratories, Hercules, CA, United States). Proteins (20 μg) were separated using 12% SDS-PAGE and transferred to a PVDF membrane (GE Healthcare). The membrane was probed with anti-β-catenin antibody (#8480; Cell Signaling, Danvers, MA, United States) or anti-β-actin antibody (013-24553; Fujifilm Wako Pure Chemicals, Osaka, Japan). Horseradish peroxidase-labeled anti-rabbit IgG antibody (GE Healthcare) and anti-mouse IgG antibody (GE Healthcare) were used as the secondary antibodies. The proteins were detected using an ImmunoStar LD chemiluminescence detection kit (Fujifilm Wako Pure Chemicals) and visualized with a LAS-1000 Lumino Image analyzer (Fujifilm, Tokyo, Japan).

### Immunocytochemistry

Cells were fixed with 4% paraformaldehyde and blocked with blocking buffer (1 × PBS/5% goat serum/0.3% Triton X-100). Cells were labeled with anti-active-β-catenin (8E7, Merck Millipore, Billerica, MA, United States) at 4°C overnight. After washing the cells, cells were incubated with secondary antibodies (Alexa Fluor 555 anti-mouse IgG, Thermo Fisher Scientific) at room temperature for 1 hr. After washing the cells, active-β-catenin was observed under the fluorescence microscope (EVOS Cell Imaging System, Thermo Fisher Scientific).

### Cell Growth

HaCaT cells (1.0 × 10^4^ cells) were seeded onto a 96-well plate (Becton Dickinson, Franklin Lakes, NJ). After 6 h, polyphenols (10 μM) were added to the wells. HaCaT cells were treated everyday with 10 μM of polyphenols, and cell proliferation was monitored using a Cell Counting Kit-8 (Dojindo, Kumamoto, Japan).

### Gene Knockdown Using shRNA

The oligonucleotides (sh-hTERT1 top: 5′-*GATCCCCGCTCGTGGAGACCATCTTTCTTTCGAAGAGAGA AAGATGGTCTCCACGA*-3′, sh-hTERT1 bottom: 5′-*AGCTTAAAAAGCTCGTGGAGACCATCTTTCTCTCTTCGAA AGAAAGATGGTCTCCA*-3′; sh-hTERT2 top: 5′-*GATCCCCGGAAGAGTGTCTGGAGCAAGTTTCGAAGAGAC TTGCTCCAGACACTCTT*-3′, sh-hTERT2 bottom: 5′-*AGCTTAAAAAGGAAGAGTGTCTGGAGCAAGTCTCTTCGAA ACTTGCTCCAGACACT*-3′) containing siRNA-expressing sequences targeting *hTERT* were cloned into the pSUPER.retro vector, as described previously (Harada et al., 2014, 2016). Viral supernatants were produced after transfection of 293T cells with pGag-pol, pVSV-G, and individual expression vectors (pSUPER.retro-sh-hTERT1, pSUPER.retro-sh-hTERT2, or pSUPER.retro-scramble shRNA) using the HilyMax reagent (Dojindo), as previously described (Yamashita et al., 2014). The target cells were infected with this viral supernatant for 24 h at 37°C. After infection, the cells were selected with 3 μg/mL puromycin (Enzo Life Sciences, Farmingdale, NY, United States) for 3 days.

### Investigation of Hair Growth in Experimental Animals

Six-week-old male C57BL/6 mice were obtained from Clea Japan (Tokyo, Japan) and allowed to adapt for a week, with food and water provided *ad libitum*. The dorsal skin of the mice was shaved with an electrical shaver at seven weeks of age; at this stage of growth, all of the hair follicles were synchronized in the telogen stage (Kim et al., 2014). Then, 0.05 mL of a 0.1% solution of polyphenol in 50% ethanol was applied and made fit in by spatula topically every day, for 35 days, and hair growth was evaluated. All mouse experiments and protocols were in accordance with the Guide for the Care and Use of Laboratory Animals, and were approved by the Ethics Committees on Animal Experimentation (Kyushu University; approval number: A28-077-0).

### Immunohistological Analysis

Skin tissue samples were fixed in 10% formalin buffer. Then, the fixative was removed using running water for 1 h. Skin pieces were dehydrated in ethanol, immersed in xylene, infiltrated with paraffin, and embedded in paraffin blocks. Paraffin-embedded hair follicles were sectioned into 5-μm-thick sections, which were stained with hematoxylin and eosin. For immunohistochemical analysis, tissue sections were deparaffinized, rehydrated, and soaked in 1 × HistoVT One (Nacalai Tesque, Kyoto, Japan). The sections were then heated at 90°C for 20 min to activate antigens. After washing with 0.1% Tween 20/TBS, tissues were blocked with Blocking One Histo (Nacalai Tesque) for 1 h at room temperature. Tissues were first stained with primary antibodies (anti-Ki-67, #12202, Cell Signaling Technology; anti-TERT, NB100-317, Novus Biologicals, Co., United States; anti-active-β-catenin, Merck Millipore, and anti-CD34, ab81289, Abcam, Cambridge, United Kingdom), and subsequently with secondary antibodies (Alexa Fluor 555 anti-rabbit IgG, Alexa Fluor 555 anti-mouse IgG or Alexa Fluor 488 anti-mouse, Thermo Fisher Scientific). After staining with Vesctashield mounting medium (Vector Laboratories, Burlingame, CA, United States), tissue samples were observed under a confocal laser-scanning microscope (FV1000, Olympus, Tokyo, Japan).

### Statistical Analysis

All experiments were performed at least 3 times, and the corresponding data are shown. The results are presented as mean ± standard deviation. Statistical significance was determined using a two-sided Student’s *t*-test. Statistical significance was defined as *P* < 0.05 (^∗^*P* < 0.05; ^∗∗^*P* < 0.01; ^∗∗∗^*P* < 0.001).

## Results

### Screening for Polyphenols That Activate hTERT Transcription in HaCaT Cells

**FIGURE 1:**
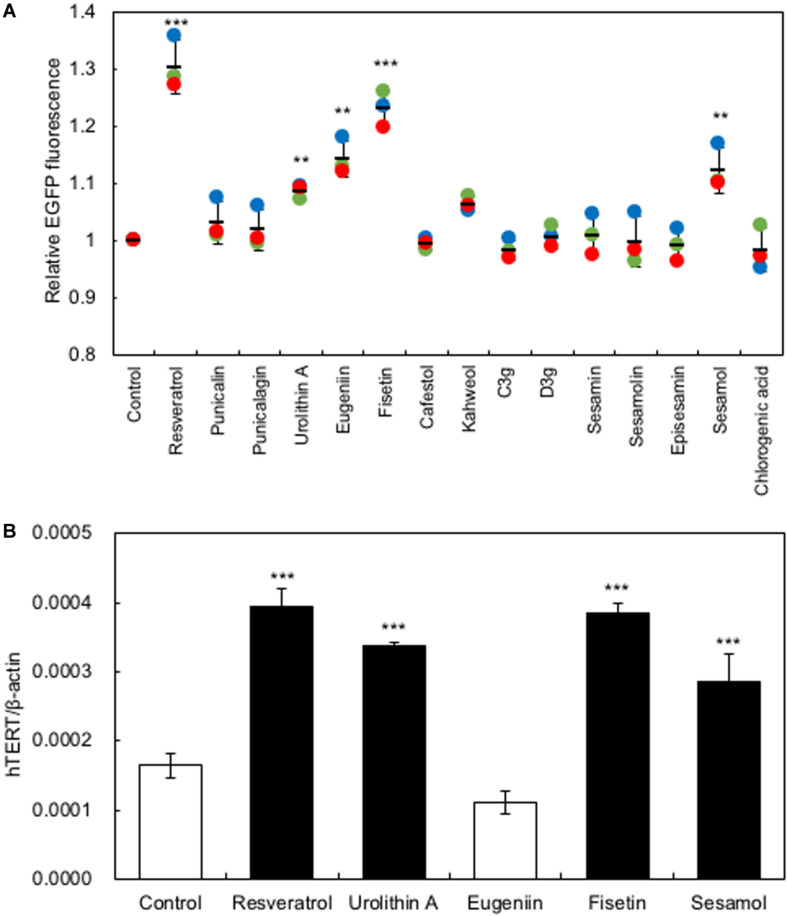
Screening for polyphenols that activate *hTERT* transcription in HaCaT cells. **(A)** Screening for polyphenols that activate *hTERT* transcription. Polyphenols (10 μM) were added to the HaCaT(hTERTp-EGFP) cells and cultured for 48 h; then, changes in EGFP fluorescence were monitored using an IN Cell Analyzer 1000. **(B)** The effect of polyphenols on the expression of endogenous *hTERT* in HaCaT cells was evaluated by qRT-PCR. Statistical significance was determined using a two-sided Student’s *t*-test. Statistical significance was defined as *P* < 0.05 (**P < 0.01; ***P < 0.001).

In the present study, we used a system to screen polyphenols that activate *hTERT* expression in recombinant HaCaT cells expressing the *EGFP* gene under the control of the *hTERT* promoter [HaCaT (hTERTp-EGFP)]. Firstly, we investigated the optimal concentration of polyphenols to augment *hTERT* expression by *in vitro* study, then used 10 μM of polyphenols for further experiments. Polyphenols (10 μM) were added to the HaCaT(hTERTp-EGFP) cells and cultured for 48 h; then, changes in EGFP fluorescence were monitored using an IN Cell Analyzer 1000 to identify polyphenols that enhance *hTERT* transcription. As Figure 1A shows, treatment with several polyphenols, including resveratrol, urolithin A, eugeniin, sesamol, and fisetin, resulted in significantly increased levels of EGFP fluorescence, indicating that these polyphenols activated the *hTERT* promoter (Figure 1A). Next, we performed qRT-PCR to test for the effect of these polyphenols on the expression of endogenous *hTERT* in HaCaT cells. We found that the polyphenols, except eugeniin, augmented the expression of *hTERT* in HaCaT cells (Figure 1B).

### Effects of Polyphenols on the Expression of Cytokine-Encoding Genes in HaCaT Cells

**FIGURE 2:**
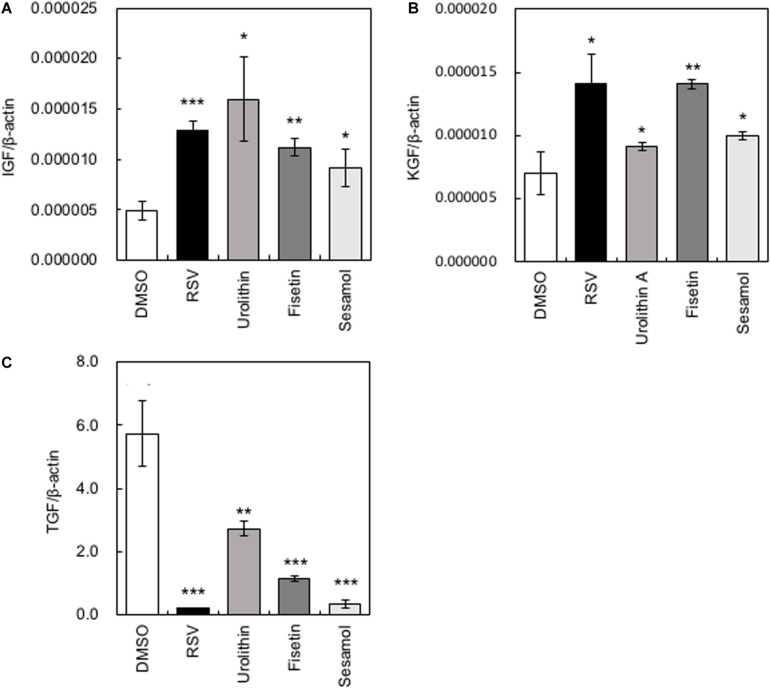
Effects of polyphenols on the expression of cytokine-encoding genes in HaCaT cells. After HaCaT cells were treated with polyphenols, the gene expression levels of *IGF-1* **(A)**, *KGF* **(B)**, and *TGF-*β*1* **(C)** were measured by qRT-PCR. Statistical significance was determined using a two-sided Student’s *t*-test. Statistical significance was defined as *P* < 0.05 (*P < 0.05; **P < 0.01; ***P < 0.001).

Several growth factors have been reported to be related to the hair growth cycle (Rho et al., 2005). We measured the gene expression levels of *Insulin-like growth factor* (*IGF-1*), *Keratinocyte growth factor* (*KGF*), and *Transforming growth factor*β*1* (*TGF-*β*1*). When HaCaT cells were treated with polyphenols, the gene expression levels of *IGF-1* and *KGF* increased significantly, while those of *TGF-*β*1* decreased significantly (Figure 2). Among the polyphenols, urolithin A showed relatively low effects on the expression of *KGF* and *TGF-*β*1*; therefore, we omitted urolithin A from subsequent experiments.

### Effects of Polyphenols on β-Catenin Activity

**FIGURE 3:**
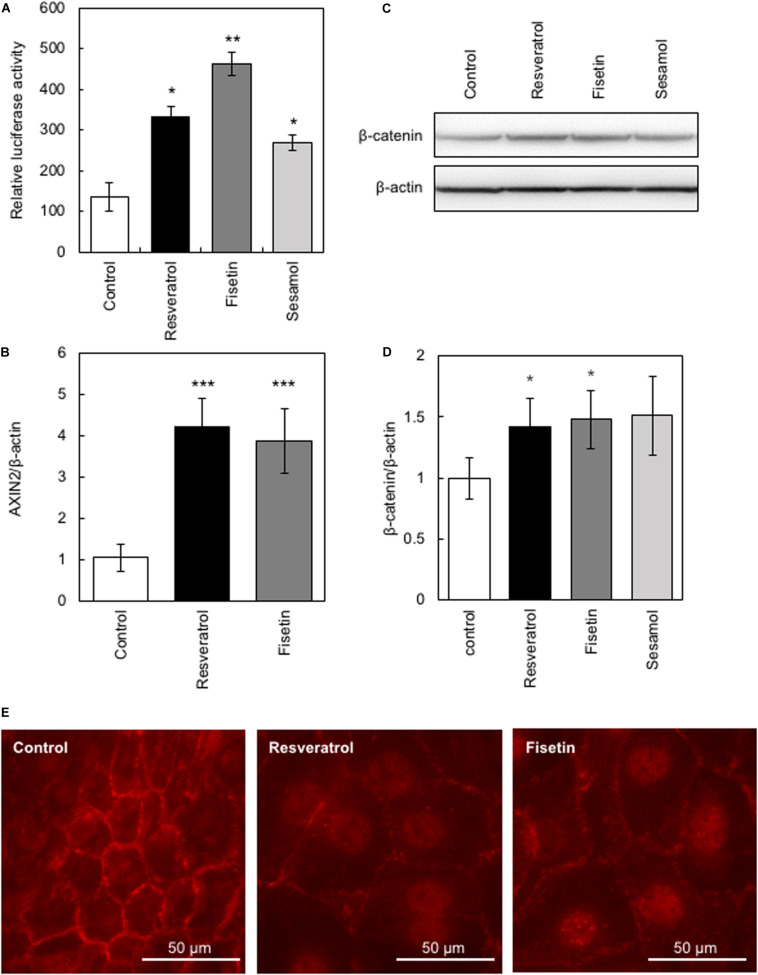
Effects of polyphenols on β-catenin activity. The effect of these polyphenols on the activity of β-catenin was evaluated. **(A)** The TOP-Flash reporter assay was performed to evaluate β-catenin activity in HaCaT cells treated with polyphenols. **(B)** The effect of polyphenols on AXIN2 expression was evaluated by qRT-PCR. **(C,D)** The effect of polyphenols on protein expression of β-catenin was evaluated by western blotting using anti-β-catenin antibody. Band intensities were quantitatively determined using ImageJ software. **(E)** The effect of polyphenols on activity of β-catenin was evaluated by immunofluorescence study using anti-active β-catenin antibody. Statistical significance was determined using a two-sided Student’s *t*-test. Statistical significance was defined as *P* < 0.05 (*P < 0.05; **P < 0.01; ***P < 0.001).

The β-catenin signaling pathway has been reported to be involved in the transcriptional regulation of *hTERT* and hair growth regulation (Shin et al., 2014); therefore, we evaluated the effects of the present polyphenols on the activity of β-catenin using the TOP-Flash reporter assay. Results clearly showed that polyphenols significantly augmented β-catenin activity (Figure 3A). Next, we evaluated the effects of these polyphenols on the protein expression and activity of β-catenin by western blotting and immunofluorescence study, respectively. Results showed that resveratrol and fisetin significantly increased the β-catenin expression (Figures 3C,D), and activated β-catenin through inducing nuclear transport (Figure 3E). Furthermore, these polyphenols augmented the expression of *AXIN2*, downstream target of β-catenin (Figure 3B). These findings collectively demonstrate that resveratrol and fisetin activate the β-catenin pathway.

### Effects of Polyphenols on the Growth of HaCaT Cells

**FIGURE 4:**
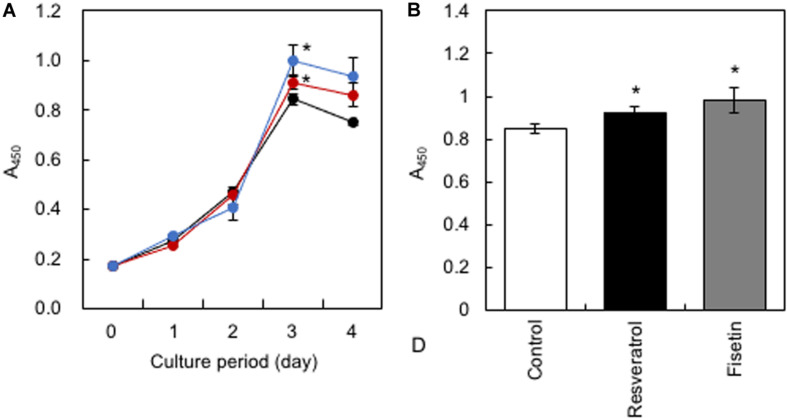
Effect of polyphenols on the growth of HaCaT cells. **(A)** After HaCaT cells were treated with 10 μM of polyphenols, cell proliferation was monitored using the Cell Counting Kit-8. **(B)** The proliferation index on the 3rd day was determined. Statistical significance was determined using a two-sided Student’s *t*-test. Statistical significance was defined as *P* < 0.05 (*P < 0.05).

HaCaT cells were treated daily with 10 μM of polyphenols, and cell proliferation was monitored using the Cell Counting Kit-8. Results showed that resveratrol and fisetin significantly increased the growth of HaCaT cells (Figures 4A,B).

### Role of hTERT in the Polyphenol-Induced Effects on HaCaT Cells

**FIGURE 5:**
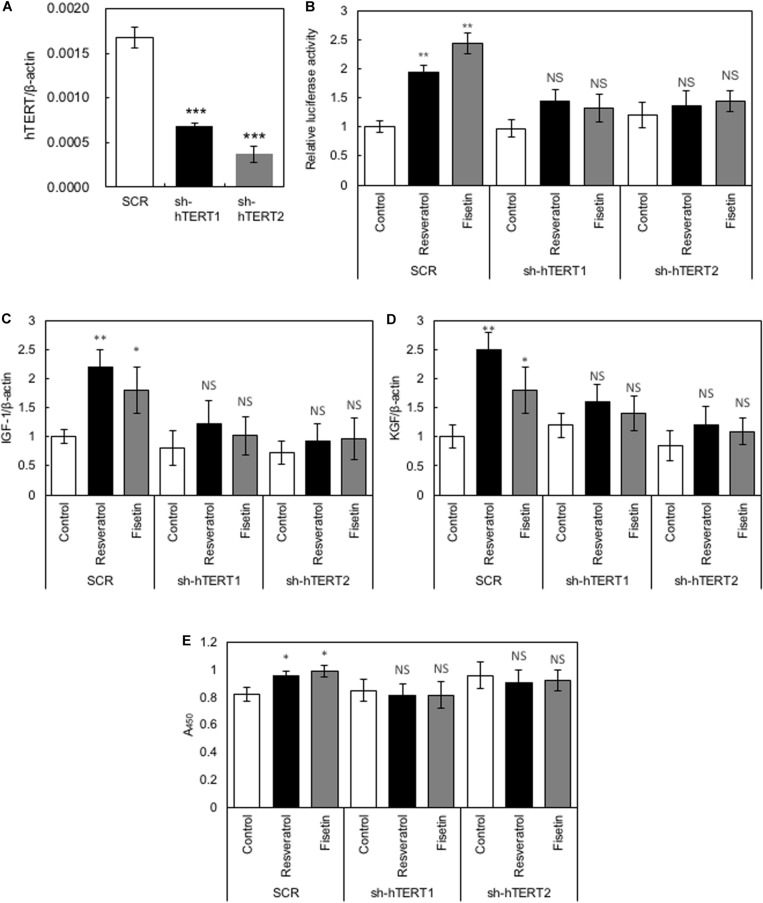
Role of hTERT in the polyphenol-induced effects on HaCaT cells. **(A)** Relative expression levels of *hTERT* in HaCaT cells transduced with retroviruses expressing shRNA targeting *hTERT* (sh-hTERT1 and 2) and scramble shRNA (SCR) were evaluated by qRT-PCR. HaCaT cells whose *hTERT* expression was reduced by shRNA (sh-hTERT-1 and 2) were treated with polyphenols, and relative β-catenin activity **(B)**, cytokine gene expression **(C,D)**, and cell proliferation **(E)** was determined. Statistical significance was determined using a two-sided Student’s *t*-test. Statistical significance was defined as *P* < 0.05 (*P < 0.05; **P < 0.01; ***P < 0.001).

To elucidate whether hTERT plays a role in the polyphenol-induced effects on HaCaT cells, we generated recombinant HaCaT cells in which the expression of *hTERT* was downregulated using shRNA (sh-hTERT-1 and sh-hTERT2). As shown in Figure 5A, these recombinant HaCaT cell lines showed significantly reduced expression of *hTERT* compared with that in control HaCaT cells transduced with scramble shRNA (SCR). Then, we tested the effects of polyphenols, including resveratrol and fisetin, on the β-catenin activity, cytokine gene expression and cell growth by using these recombinant cell lines. The results clearly showed that polyphenol-induced activation of β-catenin activity (Figure 5B), enhancement of cytokine gene expression (Figures 5C,D) and growth enhancement in HaCaT cells (Figure 5E) were abrogated by the knockdown of *hTERT*. These finding indicate that hTERT is involved in the polyphenol-induced effects on HaCaT cells.

### Promotion of Hair Growth by Polyphenols in Mice

**FIGURE 6:**
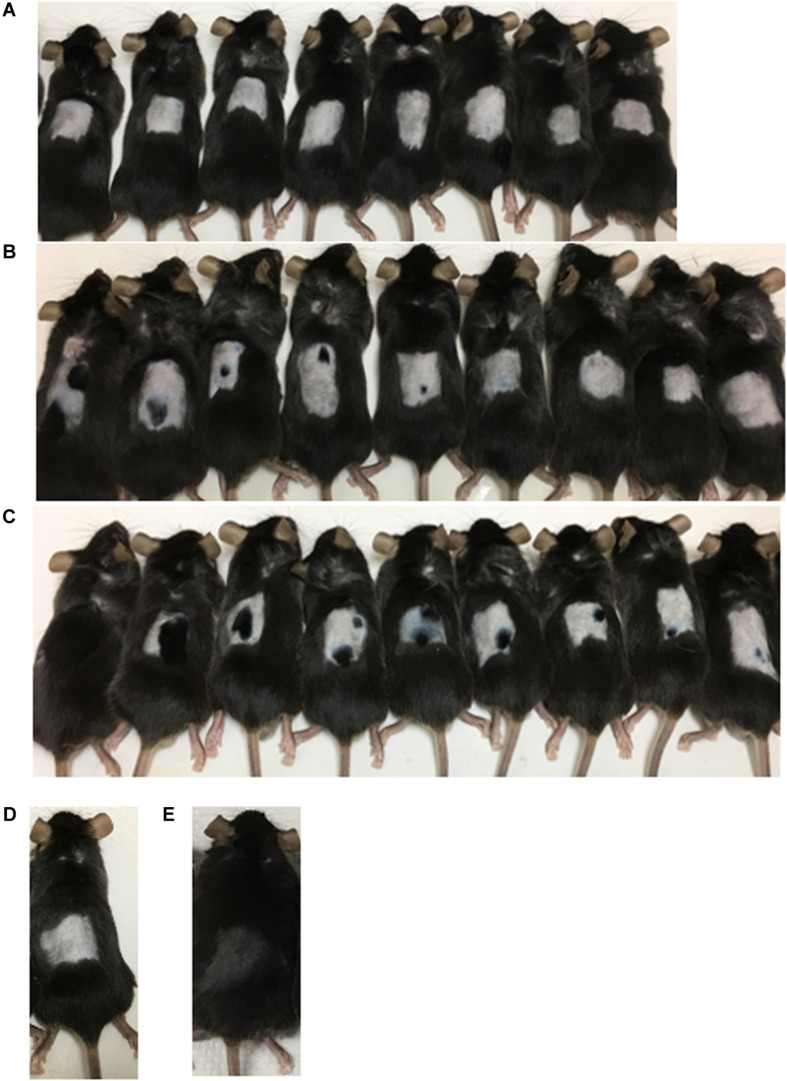
Promotion of hair growth by polyphenol treatment in mice. After the application of polyphenols on the dorsal skin of C57BL/6 mice for 35 days, the effect on hair growth was evaluated (**A**, Control; **B**, Resveratrol; **C**, Fisetin; **D**, Telogen phase; **E**, Anagen phase).

The C57BL/6 mice were divided into three groups (control, resveratrol, and fisetin). Experiments to evaluate enhancement of hair growth were performed as described in the Materials and Methods. After treatment for 35 days, the effects of polyphenols on hair growth were evaluated (Figure 6). First, mice treated with a solvent control did not show enhanced hair growth, indicating that the telogen phase was maintained for 35 days in the mice even after they were shaved. The results showed that the polyphenols enhanced hair growth in the mice (Figures 6B,C). In particular, fisetin strongly activated hair growth. All nine mice showed enhanced hair growth upon treatment with fisetin (Figure 6C); in comparison, only five of the nine mice in the resveratrol treatment group showed enhanced hair growth (Figure 6B). The difference in the hair growth rate in the experimental group might be caused by the variation in application of polyphenols. These results suggest that polyphenols, in particular fisetin, activated the transition from telogen to anagen phase, and therefore promoted hair growth.

### Effects of Polyphenols on the Skin Tissue of Mice

**FIGURE 7:**
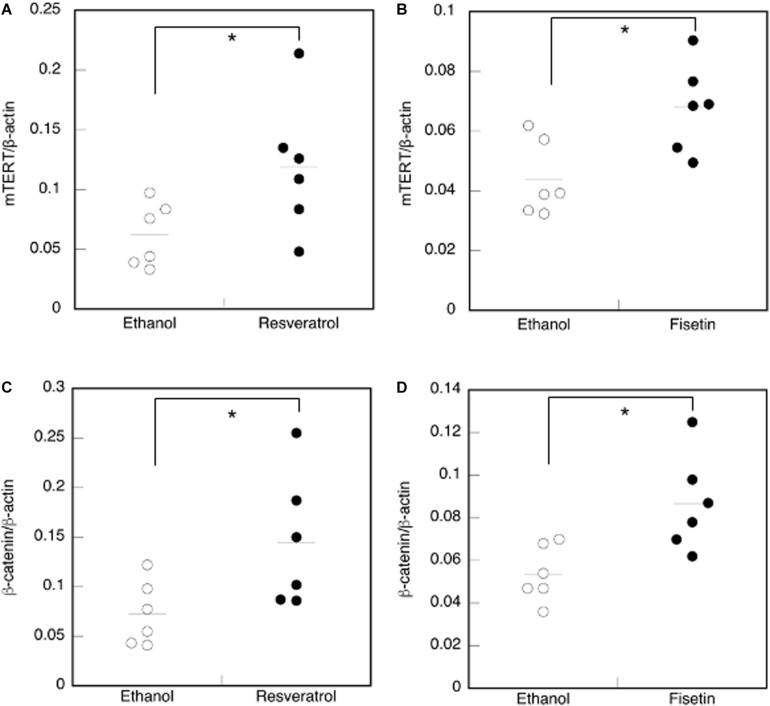
Effect of polyphenols on the expression of *mTERT* and β*-catenin* in the dorsal skin cells of mice. The expression of the *mTERT* **(A,B)** and β*-catenin* genes **(C,D)** in the dorsal skin cells of mice treated with polyphenols was investigated by qRT-PCR. Statistical significance was determined using a two-sided Student’s *t*-test. Statistical significance was defined as *P* < 0.05 (*P < 0.05).

**FIGURE 8:**
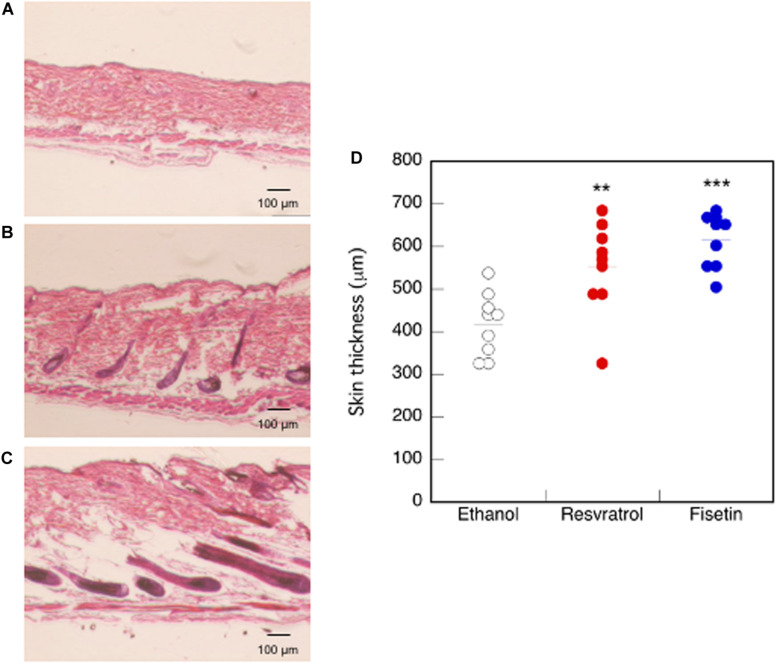
Effect of polyphenols on the dorsal skin of mice. After 35 days of treatment, histological analysis of dorsal skin was performed after H&E staining (**A**, Control; **B**, Resveratrol; **C**, Fisetin). **(D)** The length between epidermis and subcutaneous layer at the dorsal skin was quantitatively determined using ImageJ. Statistical significance was determined using a two-sided Student’s *t*-test. Statistical significance was defined as *P* < 0.05 (**P < 0.01; ***P < 0.001).

We examined mouse *TERT* (*mTERT*) and β*-catenin* gene expression in the dorsal skin cells of mice treated with polyphenols. Results showed that the *mTERT* (Figures 7A,B) and β*-catenin* (Figures 7C,D) gene expressions in these cells were significantly augmented in mice treated with resveratrol and fisetin compared with that in mice treated with the control. Therefore, the present polyphenols augment *mTERT* and β*-catenin* gene expressions in the dorsal skin cells of mice as well as in the human keratinocyte cell line, HaCaT. Histological analysis at 35 days revealed that hair follicles from control mice had remained in the telogen phase, whereas those from mice treated with resveratrol and fisetin had entered anagen (Figures 8A–C). In addition, the hair follicles in dorsal skin of mice treated with resveratrol and fisetin were found to be significantly grown (Figure 8D). Results suggest that resveratrol and fisetin induce a shift from telogen to anagen in the hair follicle.

**FIGURE 9:**
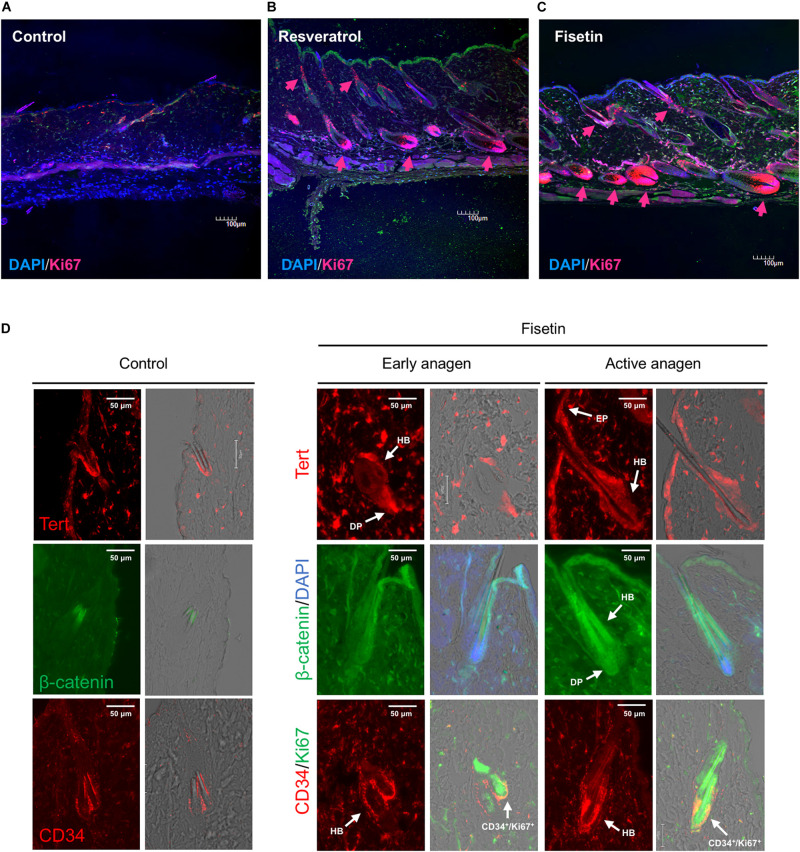
Effect of treatment with polyphenols on the proliferation of cells and expression of marker proteins in the skin sections with hair follicles. After 35 days of treatment, immunohistological analysis of skin sections was performed using anti-Ki-67 antibody and DAPI staining (**A**, Control; **B**, Resveratrol; **C**, Fisetin; arrow head; Ki67^+^ cells). **(D)** Immunohistological analysis of skin sections treated with fisetin at early anagen and active anagen were performed using anti-Tert antibody, anti-active-β-catenin antibody and anti-CD34 antibody (DP, dermal papilla; Mx, matrix; EP, epithelium). Immunohistological analysis of non-treated skin sectons were also shown (Control).

Next, we attempted to determine whether *TERT* induction in epidermis enhances the formation of hair follicle; to this end, we measured the proliferation of cells in skin sections containing hair follicles. On the dorsal skin of mice treated with resveratrol (Figure 9B) and fisetin (Figure 9C), and non-treated mice (Figure 9A), a number of proliferating cells (Ki67^+^ cells, arrow head) were observed around hair matrix and around outer/inner root sheath (ORS/IRS). These results indicate that *hTERT* induction in the epidermis triggers the proliferation of cells such as hair follicle dermal papilla, hair matrix cells and ORS/IRS, which results in the formation of hair follicle and promotion of hair growth in mice treated with *hTERT*-augmenting polyphenols.

Finally, we tested the expression of marker proteins (Tert, β-catenin and CD34) in the skin section treated with fisetin during the process of hair growth. Early anagen was detected 2–3 weeks after the treatment with fisetin, and active anagen was formed about another week after its emergence. The expression of marker proteins in non-treated skin sections were also shown in Figure 9D. Tert was strongly expressed in skin epithelium (EP), hair bulge (HB) and dermal papilla (DP) at early anagen, and showed a pan-epithelial expression at active anagen. β-catenin was consistently detected in ORS, IRS, hair shaft and DP during the course of hair growth, but its expression in DP clearly increased at active anagen. CD34, a cell membrane marker of hair follicle stem cells, was highly detected in the cells surrounding HB at early and active anagen (Figure 9D). CD34^+^/Ki67^+^ cells were found around HB region, suggesting that hair follicle stem cells were vigorously proliferating. Although CD34^+^ cells can be detected in the skin section of control mice, the number of CD34^+^ cells increased in the skin sectin of fisetin-treated mice.

## Discussion

### Identification of Polyphenols That Augment hTERT Transcription

In the present study, we used a reporter HaCaT cell line that expressed *EGFP* under the control of the *hTERT* promoter in order to identify polyphenols that activate *hTERT* transcription. We identified two polyphenols, namely resveratrol and fisetin that promoted hair growth in experimental mice. Although many researchers have identified polyphenols that regulate *hTERT* transcription (Chen et al., 2017), many of these have been studied in the context of downregulation of *hTERT* transcription in cancer cells. We have previously reported that resveratrol activates *hTERT* transcription in normal human umbilical cord fibroblast cells (Yamashita et al., 2012). These results suggest that *hTERT* transcription is regulated by polyphenols in a manner that is dependent upon cell type and context.

In this study, resveratrol and fisetin showed strong hair-growth-promoting activity. Resveratrol and fisetin are natural polyphenolic compounds and have many pharmacological and physiological activities (Bai et al., 2018). Recent studies have shown that both polyphenols are known to be a potent activator of sirtuin, a longevity gene, and calorie-restriction mimetics, suggesting that these polyphenols can alleviate aging-related functional decline in organisms (Villalba and Alcaín, 2012). The hair-growth-promoting activities of resveratrol and fisetin are recognized as novel anti-aging effects of these polyphenols. In a future study, we will seek to elucidate the structure-function relationships of these polyphenols and identify the structural features of polyphenol that activate *hTERT* transcription.

### Involvement of Cytokines in Polyphenol-Induced Hair Growth Promotion

Various cytokines, including IGF-1, KGF, and TGF-β1, play essential roles in hair growth. IGF-1 plays a critical role in regulating cellular proliferation and migration during hair follicle development, while KGF stimulates hair follicle proliferation. In contrast, TGF-β1 is involved in the regulation of hair follicle regression by inducing apoptosis and inhibiting keratinocyte proliferation and in the maintenance of niche of hair follicle stem cells (Mesa et al., 2015). Several plant extracts promote hair growth by regulating the expression of hair-growth-related cytokines (Kim et al., 2014; Shin et al., 2016). In the present study, resveratrol and fisetin increased the expression of IGF-1 and KGF, and decreased that of TGF-β1, in HaCaT cells, thereby promoting hair growth. In future studies, we aim to clarify the mechanisms underlying polyphenol-induced regulation of cytokine expression.

### Involvement of β-Catenin in Polyphenol-Induced Hair Growth Promotion

Numerous reports have described the interaction between Wnt/β-catenin and hTERT. Zhang and Hoffmeyer et al. have shown that hTERT is a novel target of the Wnt/β-catenin pathway (Hoffmeyer et al., 2012; Zhang et al., 2012). In contrast, Park et al. (2009) reported that hTERT functions as a transcriptional modulator of the Wnt/β-catenin signaling pathway. These results suggest a close link between the Wnt/β-catenin signaling pathway and hTERT. The present results clearly demonstrate that the *hTERT*-augmenting polyphenols, namely resveratrol and fisetin, activated β-catenin expression, and that polyphenols activated β-catenin in a manner dependently upon hTERT. Considering together with that β-catenin is involved in various stages of hair morphogenesis, induction of hair follicles and hair growth promotion (Gat et al., 1998; Närhi et al., 2008; Bejaoui et al., 2020), this polyphenol-induced activation of β-catenin via *hTERT* augmentation might be a key event for hair growth promotion.

### Molecular Basis for Hair-Growth-Promotion

Various plant extracts and chemicals with hair-growth-stimulating effects have been reported. Although precise underlying mechanisms remain unknown, these extracts and chemicals are considered to elicit these effects by accelerating blood flow, inducing a shift from telogen to anagen, mediating activation of the dermal papilla, inhibiting dihydrotestosterone, and exerting anti-inflammatory effects (Junlatat and Sripanidkulchai, 2014; Kim et al., 2014; Park et al., 2017; Shen et al., 2018; Zhang et al., 2019; Zhou et al., 2020). The hair follicle contains several different types of cells; among these, the dermal papilla cells are major components of hair that play a critical role in inducing anagen phase and maintaining hair growth. Several growth factors, including IGF-1, KGF, and TGF-β, are known to modulate the proliferation of the follicular epithelium, and control follicle development and cytokine expression in hair cells. In addition, The Wnt/β-catenin pathway is known to be important for the initiation, development, and growth of hair follicles as well as for the induction of anagen (Gat et al., 1998; Park et al., 2012). Several molecules, cells and signaling pathways are known to be involved in hair growth promotion, β-catenin was conceivable as a key molecule of *TERT*-augmenting polyphenol to promote hair growth. In our study, fisetin induced Tert in epidermis, which resulted in the proliferation of ORS/IRS and Mx, activation of hair follicle stem cells (CD34^+^ cells) and β-catenin, which might resulted in the formation of hair follicle and promotion of hair growth. In future studies, we aim to clarify the whole picture of molecular mechanisms of *TERT*-augmenting polyphenol to promote hair growth.

## Data Availability Statement

The raw data supporting the conclusions of this article will be made available by the authors, without undue reservation, to any qualified researcher.

## Ethics Statement

The animal study was reviewed and approved by the Ethics Committees on Animal Experimentation (Kyushu University).

## Author Contributions

CK, MO, and NU performed the experiments and collected the data. YK developed the study design and wrote the manuscript. All authors contributed to the article and approved the submitted version.

## Conflict of Interest

The authors declare that the research was conducted in the absence of any commercial or financial relationships that could be construed as a potential conflict of interest.
